# Mycorrhizal status and host genotype interact to shape plant nutrition in field grown maize (*Zea mays* ssp. *mays*)

**DOI:** 10.1007/s00572-023-01127-3

**Published:** 2023-10-18

**Authors:** Meng Li, Sergio Perez-Limón, M. Rosario Ramírez-Flores, Benjamín Barrales-Gamez, Marco Antonio Meraz-Mercado, Gregory Ziegler, Ivan Baxter, Víctor Olalde-Portugal, Ruairidh J. H. Sawers

**Affiliations:** 1 Department of Plant Science, The Pennsylvania State University, State College, PA 16802 USA; 2 Departamento de Biotecnología y Bioquímica, Centro de Investigación y de Estudios Avanzados (CINVESTAV-IPN), Irapuato, Guanajuato, 36821 México; 3 Bioscience Division, Oak Ridge National Laboratory, 1 Bethel Valley Rd, Oak Ridge, TN 37830 USA; 4 Postgrado en Recursos Genéticos y Productividad-Genética, Campus Montecillo, Colegio de Postgraduados, Montecillo, Texcoco, Edo. de México 56230 México; 5 Donald Danforth Plant Science Center, St. Louis, MO 63132 USA

**Keywords:** Arbuscular mycorrhizal fungi, Maize, Ionome, Quantitative trait loci

## Abstract

Arbuscular mycorrhizal fungi (AMF) establish symbioses with the major cereal crops, providing plants with increased access to nutrients while enhancing their tolerance to toxic heavy metals. However, not all plant varieties benefit equally from this association. In this study, we used quantitative trait loci (QTL) mapping to evaluate the combined effect of host genotypic variation (G) and AMF across 141 genotypes on the concentration of 20 mineral elements in the leaves and grain of field grown maize (*Zea mays* spp. *mays*). Our mapping design included selective incorporation of a *castor* AMF-incompatibility mutation, allowing estimation of AMF, QTL and QTLxAMF effects by comparison of mycorrhizal and non-mycorrhizal plants. Overall, AMF compatibility was associated with higher concentrations of boron (B), copper (Cu), molybdenum (Mo), phosphorus (P), selenium (Se) and zinc (Zn) and lower concentrations of arsenic (As), iron (Fe), magnesium (Mg), manganese (Mn), potassium (K) and strontium (Sr). In addition to effects on individual elements, pairwise correlation matrices for element concentration differed between mycorrhizal and non-mycorrhizal plants. We mapped 22 element QTLs, including 18 associated with QTLxAMF effects that indicate plant genotype-specific differences in the impact of AMF on the host ionome. Although there is considerable interest in AMF as biofertilizers, it remains challenging to estimate the impact of AMF in the field. Our design illustrates an effective approach for field evaluation of AMF effects. Furthermore, we demonstrate the capacity of the ionome to reveal host genotype-specific variation in the impact of AMF on plant nutrition.

**Supplementary Information:**

The online version contains supplementary material available at 10.1007/s00572-023-01127-3.

## Introduction

Arbuscular mycorrhizal fungi (AMF) are a widely distributed group of soil microbes that form mutualistic associations with more than 80% of terrestrial plant species, including the majority of staple crops (Wang and Qiu 2006; Cobb et al. 2021; Thirkell et al*.* 2022). Arbuscular mycorrhizal (AM) symbiosis connects plant roots to an extensive network of root-external fungal hyphae, dramatically enhancing the potential for nutrient foraging in the soil. One of the major benefits of AM symbiosis to hosts is enhanced access to soil phosphorus (P), with AMF providing up to 90% of the total P taken up by a plant (van der Heijden et al. 2015). AM symbiosis also contributes to the uptake of other essential nutrients such as nitrogen (N), sulphur (S), zinc (Zn) and copper (Cu), as well as mitigating the toxicity of heavy metals by reducing both movement into the roots and translocation to the shoot (Bhandari and Garg 2017; Lehmann and Rillig 2015; Riaz et al. 2021; Ruytinx et al. 2020).

To characterize the diverse effects of AMF on host mineral nutrition, researchers have profiled the ionome—or complete set of mineral nutrients (Baxter 2015; Gerlach et al. 2015; Lahner et al. 2003; Ramírez-Flores et al*.* 2017). The technique of inductively coupled plasma mass spectrometry (ICP-MS) has proven especially useful in simultaneously measuring the concentrations of multiple elements in a single sample, although standard protocols do not allow quantification of N. Ionome profiling of plants grown with or without inoculation with AMF in the greenhouse has identified strong element specific effects of AM symbiosis on host plant mineral nutrition (Gerlach et al. 2015). Furthermore, analysis of diverse host varieties has shown that covariance between the concentrations of different elements responds to AM symbiosis (Ramírez-Flores et al. 2017). These results indicate that not only do plant varieties differ in their capacity to acquire nutrients but that the impact of AM symbiosis on this capacity also differs. Here, we build on this observation by using genetic mapping in field-grown maize (corn; *Zea mays* ssp. *mays*) to test the hypothesis that the genetic architecture of element concentration is indeed distinct between mycorrhizal (M) and non-colonized (NC) plants. Understanding this genetic architecture will provide valuable insights for breeding and agricultural practices aimed at optmizing crop nutrient uptake (Berruti et al. 2015), reducing levels of toxic heavy metals (Göhre and Paszkowski 2006; Gupta et al. 2022) or biofortifying crops to enhance the nutritional value for human consumption (White and Broadley 2009).

Genetic mapping uses correlations between genetic markers and trait values in a diverse population to link regions of the genome to phenotypic variation. Continuous trait variation is typically associated with the action of many regions throughout the genome, each such region being referred to as a quantitative trait locus (QTL; Broman and Sen 2009). For any given trait, the number of associated QTLs, their relative importance and their interactions define the genetic architecture. In the context of the ionome of M and NC plants, changing patterns of element covariance imply that different QTL effects—and potentially different underlying genes—are important in the presence or absence of AM symbiosis. Physiological and molecular studies support this interpretation through definition of distinct *direct* (i.e. directly from soil to root) and *AMF* (i.e. via fungal hyphae) nutrient uptake pathways and associated molecular components (Smith et al. 2003). In the case of P, specific members of the PHT1 phosphate transporter family function in either the direct or AMF pathways, their expression and accumulation patterns responding to colonization by AMF (Bucher 2007; Chiu and Paszkowski 2019; Salvioli di Fossalunga and Novero 2019). Analogous patterns of expression, consistent with either direct or AMF uptake, also are observed in the ammonium and nitrate transporter gene families (Hui et al. 2022; Koegel et al. 2013; Wang et al. 2020), in the ZIP Zn transporter family (Nguyen et al. 2019) and in the SULTR sulphate transporter family (Casieri et al. 2012). Furthermore, the relative expression of transporter genes involved in direct or AMF pathways can differ among plant varieties (Giovannini et al. 2022; Sawers et al. 2017), consistent with pathway-specific host genetic variation and shifting patterns of element covariance in response to AM symbiosis.

**Fig. 1:**
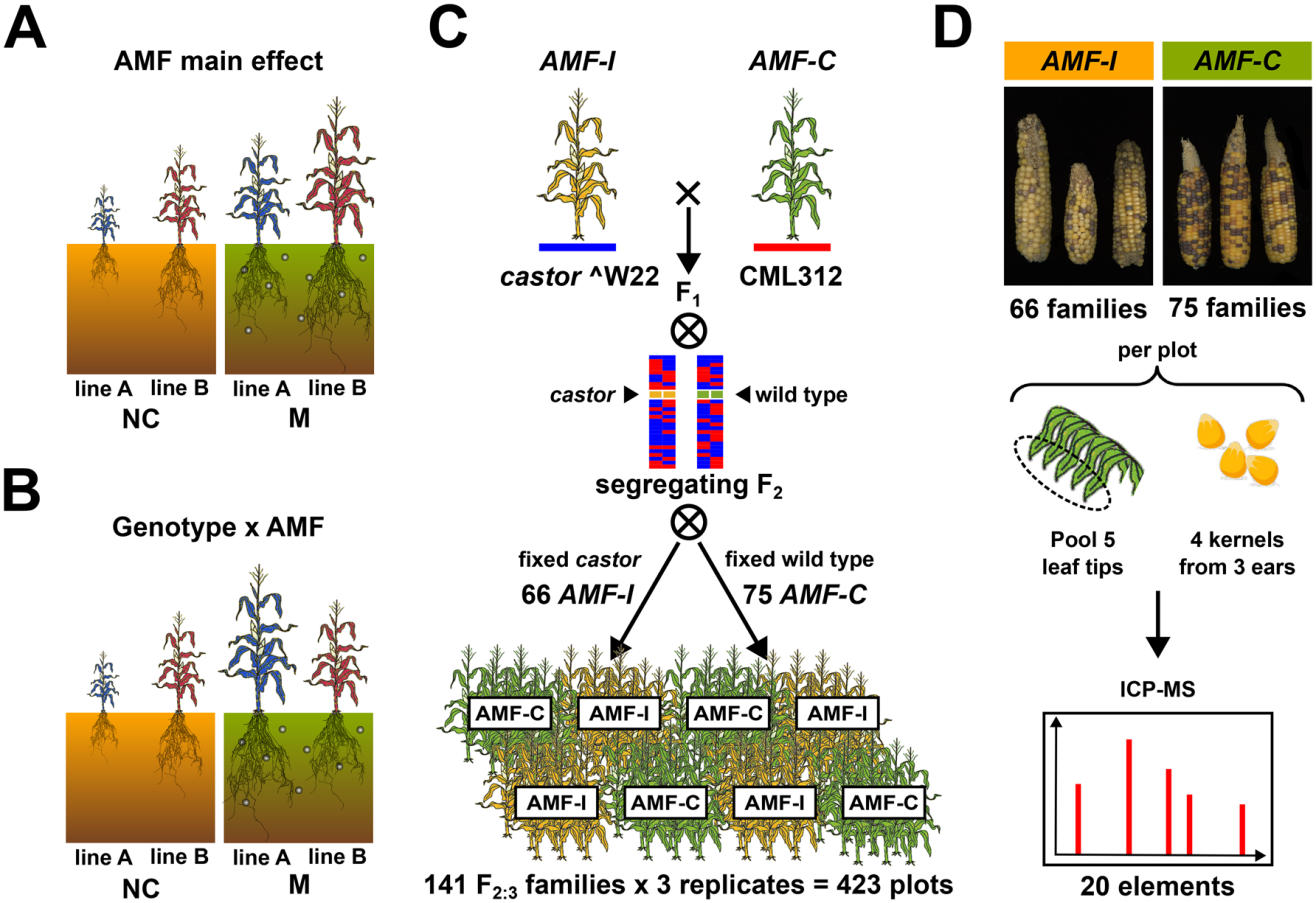
Experimental question and approach.** A** Hypothetical main effect of arbuscular mycorrhizal fungi (AMF). Mycorrhizal (M) plants perform better than those that are non-colonized (NC). The effect is additive, affecting the two plant varieties, line A and line B, equally. **B** Hypothetical interaction between AMF effect and host genotype. In contrast to (**A**), the impact of AMF differs between the two plant varieties. As shown, line B is superior in the NC condition, and line A is superior in the M condition. **C** To estimate genotype x AMF effects, the AMF incompatible (AMF-I) mutant *castor* in the W22 background was crossed to the AMF compatible (AMF-C) wild-type line CML312. The resulting F_2_ generation segregated for the *castor* mutation while mixing W22 and CML312 gene content across the genome. F_2_ individuals homozygous for *castor* (AMF-I) or the corresponding wild type allele (AMF-C) were identified by molecular genotyping and selected as parents of F_2:3_ families. In total, 66 AMF-I and 75 AMF-C F_2:3_ families were generated and evaluated in a triplicated field trial using 3-row plots. **D** To study effects on the plant ionome, leaves and grain were sampled from each plot and analysed by ICP-MS, determining the concentration of 20 different elements

To model differences in genetic architecture between M and NC plants, we can use an approach typically employed to define the genetic underpinning of so-called *genotype x environment* interaction (GxE; Des Marais and Juenger 2010). GxE refers to not all varieties responding equally to changes in their environment (Fig. 1A, B). For example, the best crop variety at sea level may not be the best at high elevation (Perez-Limón et al. 2022). Here, we consider growth with or without AM symbiosis to be two different “environments”, and we use GxAMF to describe host genotype-specific responses to AMF (e.g. Kaeppler et al. 2000; Ramírez-Flores et al. 2020; Sawers et al. 2017). Just as QTLs contribute to trait variation, QTLxE interactions describe the genetic basis of GxE. A given QTL may act in all environments (*constitutive*; i.e., no QTLxE), it may act in some environments and not others (*conditional*), or it may act in all environments but with the sign of the effect changing (*antagonistic pleiotropy*; Des Marais and Juenger 2010). This last scenario includes genetic variants that are beneficial in one environment but deleterious in another, producing a trade-off with respect to optimizing the genotype to a given environment. Previously, we have used the GxE framework to characterize the impact of AMF on variation in maize phenological traits and yield components (Ramírez-Flores et al. 2020). This approach allows partitioning trait variation into the “average” (*main*) effect of AMF on all host genotypes, the additive effect of QTLs regardless of plant AM status and GxAMF effects that are plant genetic effects modified by AM symbiosis. Here, we apply this approach to the analysis of the ionome of leaves and grain.

To evaluate the large number of plants required for genetic mapping, it is advantageous to conduct field experiments. In addition to being relatively easy to scale-up, field experiments may have greater agronomic relevance than, for example, small-pot greenhouse studies. However, evaluating AM symbiosis in the field is complicated by the difficulty of manipulating the field AMF community (Feldmann 2009; Leiser et al*.* 2016; Ryan and Graham 2018). Fungicides or AMF inoculants can be used to either reduce or augment the AMF community, but treatments may not be effective and the native community may persist (Ortas 2012; Buysens et al. 2015; Salomon et al. 2022). As an alternative to directly manipulating AMF in the soil, researchers have proposed a genetic approach through the use of AMF-incompatible mutant plants to act as controls or *sentinels* (Bowles et al. 2016; Groten et al. 2015; Rillig et al. 2019; Watts-Williams and Cavagnaro 2015). The ideal sentinel plant will fail to establish AM symbiosis but in other ways be normal. Then, the impact of AMF can be estimated by comparing the sentinel variety to wild-type individuals. This approach has been employed previously in wild tobacco (*Nicotiana attenuata*) using a *ccamk* mutant (Groten et al. 2015), and in maize, by ourselves, using a *castor* mutant (Ramírez-Flores et al. 2020). CCAMK and CASTOR are both components of the *common symbiotic signalling pathway* and have been shown to be necessary for the establishment of AM symbiosis in several plant species (Charpentier et al. 2008; Gutjahr et al. 2008; Singh and Parniske 2012). To map GxAMF effects in the field, sentinel mutations can be used to generate the non-mycorrhizal field “environment”. We have implemented this approach both previously and here using a custom biparental maize mapping population incorporating both wild-type and *castor* mutant families (Ramírez-Flores et al. 2020).

In this study, we characterize the impact of AMF on the genetic architecture of the leaf and grain ionome of field grown maize. We used a biparental genetic mapping population of 141 families, 66 of which were homozygous for the *castor* mutation and incompatible with AMF (AMF-I) and 75 of which were AMF-compatible (AMF-C) wild type (Fig. 1; Ramírez-Flores et al. 2020). By comparing AMF-C and AMF-I subpopulations, we estimated the effect of AMF on the concentration of 20 different elements. Outside of the *castor* locus, the 141 families segregated for the two parental genotypes allowing us to perform genetic mapping. By estimating the effect of AMF on genetic architecture, we identified genetic regions linked to AMF-specific parental differences in element concentration, revealing the importance GxAMF effects to the nutritional outcome of AM symbiosis.

## Materials and methods

### Plant material and experimental design

A mapping population was developed from the cross between a homozygous *castor* mutant in the temperate W22 background and a wild-type subtropical inbred line CML312, as previously described (Ramírez-Flores et al. 2020; Fig. 1). The population contained subpopulations of 75 AMF-compatible (wild-type; AMF-C) and 66 AMF-incompatible (homozygous *castor* mutant; AMF-I) maize F_2:3_ families. The experiment was conducted in Ameca, Jalisco, Mexico (20.57 N latitude, − 104.04 W longitude). The field was fertilized at planting with 250 kg/ha of diammonium phosphate (DAP; 18-46-00 NPK) and again at 40 days after planting with 250 kg/ha of urea (46-00-00, NPK) which is standard for maize grown in the area. Families were evaluated in three complete blocks. Each plot contained 45 individuals in three 2 m rows with 75 cm between rows. Only plants in the central row of each plot were evaluated to minimize shading and potential below ground impacts of adjacent plots. Plots of AMF-C and AMF-I families were alternated, the order of the families being randomized within each block. “Plot” was considered the unit of replication. Further details of the mapping population and experimental design are presented in Ramírez-Flores et al. (2020).

### Determination of element concentration by inductively coupled plasma mass spectrometry

Five flag leaves were collected per plot at flowering and oven dried at 70 °C for 48 h. After drying, 10 cm from the tip was taken from each flag leaf and pooled to obtain one sample per plot. For the grain samples, 4 kernels were selected randomly from 3 ears per plot (Fig. 1D). Element concentration was determined as described previously (Ramírez-Flores et al. 2017). Briefly, flag leaves and grain samples were analysed by ICP-MS to determine the concentration of 20 elements. Samples were digested in 2.5 mL concentrated nitric acid (AR Select Grade, VWR) with an added internal standard (20 ppb In, BDH Aristar Plus). Element concentration was measured using an Elan 6000 DRC-e mass spectrometer (Perkin-Elmer SCIEX) connected to a PFA microflow nebulizer (Elemental Scientific) and Apex HF desolvator (Elemental Scientific). A control solution was run every tenth sample to correct for machine drift both during a single run and between runs.

### Data preparation and processing

Analyses were performed in R 4.0.4 (R Core Team 2022). Data were trimmed to remove outliers beyond 1.5 × the interquartile range for each element/tissue combination as per the R/ graphics::boxplot default criteria and then adjusted on a per block basis to a spline model fitted against row number using R/stats::smooth.spline to reduce sub-block spatial variation. Mean element concentrations for each F_2:3_ family were obtained by averaging the three replicates. The differences between element concentrations of AMF-C and AMF-I families in leaves and grain were tested by Wilcoxon test with *p* values adjusted based on the number of elements using the Bonferroni adjustment method. To further explore the differences in element accumulation between AMF-C and AMF-I families, mycorrhizal responses (MR) of element accumulation were calculated for each element in leaves and grain using the equation: 

$$\text{MR} = \text{ln}(\text{AMF-C/AMF-I}).$$

### Mixed effects linear models

For each element, a mixed effects linear model was fitted using the restricted maximum-likelihood method with R/lme4::lmer, such that:

$$y_{\mathrm i\mathrm j\mathrm k\mathrm m}=\mu+B_{\mathrm i}+G_{\mathrm j}+C_{\mathrm k}+\varepsilon_{\mathrm i\mathrm j\mathrm k}$$

where the response variable *y*_ijkm_ is a function of the overall mean (*μ*)⁠, random effect of the block (*B*_i_)⁠, random effect of genotype (*G*_j_)⁠ and a fixed effect of the AMF status (AMF-I or AMF-C; *C*_k_⁠) ⁠and the residual (*ε*_ijk_). Best linear unbiased prediction (BLUP) values for the genotypic effect (G) were extracted using R/lme4::ranef. We calculated fitted values by adding BLUPs to the grand mean for data visualization and downstream analyses using natural units. Broad-sense heritability for each continuous trait was estimated based on the linear mixed model results using the *bwardr::Cullis_H2* function (Cullis et al. 2006).

### Multivariate analyses

Fitted values from the mixed effects linear models were used for multivariate analyses. Fitted values of the elements B, Na, Al, Se and Rb showed low heritable variation in either leaf or grain and were removed from the analyses. Factor analysis was conducted using the R/stats::factanal function, with the ‘varimax’ rotation method. Pairwise correlations among elements were calculated for both AMF-C and AMF-I subpopulations. Correlations between elements were calculated using R/stats::cor with the spearman correlation method. To test whether correlation matrices were equal between AMF-C and AMF-I subpopulations, a Chi-square test was performed using the R/decorate::delaneau.test (Hoffman 2021).

### QTL mapping

QTL mapping was performed as described in Ramírez-Flores et al. (2020) using R/qtl (Broman et al. 2003). The BLUPs for leaf and grain element concentration were used as phenotypes. Single-QTL standard interval mapping was run using the *scanone* function under the Haley-Knott regression model with default parameters. When included, the genotype at *castor* was treated as a covariate (AMF-C = 1; AMF-I = 0; hereafter AMF). To identify QTLxAMF interaction (Broman and Sen 2009), three model scenarios were considered for mapping: (i) two separate models for the AMF-I (*H*_0I_) and AMF-C (*H*_0C_) subpopulations, (ii) a model using the whole population with AMF as an additive covariate (*H*_a_), and (iii) a model using the whole population with AMF as an interactive covariate (*H*_f_). QTL support was expressed as logarithm of the odds (LOD) of presence/absence of a QTL at a given map position. LOD significance thresholds were established using permutation (1000 permutations, α = 0.1). Evidence for QTLxAMF interaction was obtained by inspection of the separate subpopulation analyses and by comparing *H*_a_ and *H*_f_ models, with the difference LOD_i_ = LOD_f_-LOD_a_ compared to the permutation threshold difference LOD_thr_i_ = LOD_thr_f_-LOD_thr_a_. Individual QTL were combined into a multiple-QTL model on a per phenotype basis to estimate variance explained. Where evidence was found of QTLxAMF interaction in the single-QTL scan, the interaction also was included in the multiple-QTL model. Multiple-QTL models were evaluated using the *fitqtl* function and non-significant (α = 0.1) terms removed according to the drop-one table.

## Results

### The *castor* mutation impacts the leaf and grain ionome of field grown maize

**Fig. 2:**
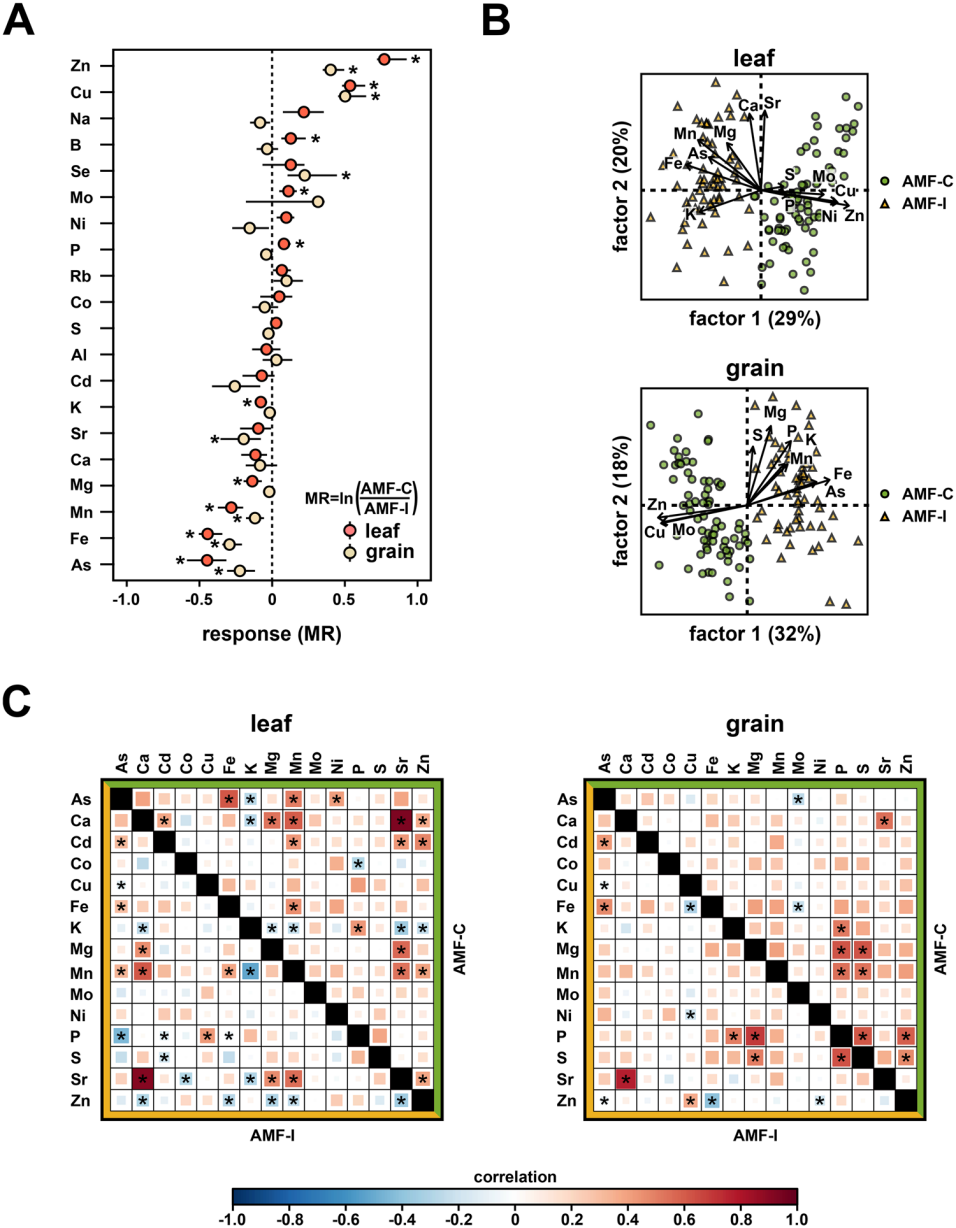
Mycorrhizal colonization affects leaf and grain element concentration in field grown maize. **A** Mycorrhizal response (MR) for element concentrations in leaf and grain. MR for each element was estimated as the natural log ratio between element concentration in compatible (AMF-C) and incompatible families (AMF-I). Error bars show 95% confidence intervals. Asterisks indicate significant differences from zero. **B** Factor analysis biplots showing element concentration patterns and loadings on the first two dimensions for leaf and grain in AMF-C (green circles) and AMF-I (yellow triangles) families. **C** Pairwise correlations in element concentration in leaf and grain for AMF-C (above the diagonal) and AMF-I (below the diagonal) subpopulations. Positive correlations are shown in red and negative correlations in blue. Significant correlations (*p* < 0.05) are marked with an asterisk

To assess the impact of AM symbiosis on maize mineral nutrition in the field, we evaluated the concentrations of 20 elements in leaf and grain samples from a previously described trial of 75 AMF-compatible (AMF-C) and 66 AMF-incompatible (AMF-I) F_2:3_ families (Fig. 1; Ramírez-Flores et al. 2020). Among the 20 tested elements, ten in leaves and six in grain differed significantly in concentration between AMF-C and AMF-I subpopulations (Fig. 2A; Table 1). AMF compatibility was associated with significantly (Bonferroni adjusted *p* < 0.05) elevated concentrations of copper (Cu), selenium (Se) and zinc (Zn), and significantly diminished concentrations of arsenic (As), iron (Fe) and manganese (Mn), in both leaves and grain. There were additional AMF-C leaf-specific significant increases in the concentrations of boron (B), molybdenum (Mo) and phosphorus (P), and reductions in magnesium (Mg) and potassium (K). Finally, the concentration of selenium (Se) was greater, and the concentration of strontium (Sr) lower, in AMF-C grain than in AMF-I grain. We performed a factor analysis with the ionome data and could distinguish AMF-C and AMF-I families by the first latent variable in both leaf and grain, an effect predominantly driven by Zn, Cu and Fe concentrations (Figs. 2B and S1).

**Table 1:** Element concentrations (mg kg^-1^) in leaves and grain of mycorrhizal compatible (AMF-C) and incompatible (AMF-I) families

	**Leaf**							**Grain**					
	**AMF-C**		**AMF-I**					**AMF-C**		**AMF-I**			
**Element**	**Mean**	**S.E.**	**Mean**	**S.E.**	***p***	**H**^**2**^	**Mean**		**S.E.**	**Mean**	**S.E.**	***p***	**H**^**2**^
Al	11	0.38	12	0.41	NS	0.51	0.57		0.018	0.55	0.02	NS	0
As	0.044	0.002	0.068	0.003	***	0.65	0.006		0.0002	0.007	0.0003	***	0.26
B	19	0.42	16	0.56	*	0.21	0.54		0.013	0.56	0.015	NS	0
Ca	6300	190	7000	210	NS	0.64	84		2.8	91	3.7	NS	0.28
Cd	0.032	0.001	0.034	0.001	NS	0.73	0.004		0.0002	0.005	0.0003	NS	0.58
Co	0.06	0.002	0.057	0.002	NS	0.53	0.004		0.0001	0.005	0.0001	NS	0.13
Cu	5.6	0.11	3.3	0.11	***	0.69	1.7		0.034	1	0.046	***	0.4
Fe	76	1.7	120	3.9	***	0.62	21		0.32	27	0.78	***	0.2
K	31,000	390	33,000	530	*	0.61	5900		76	6000	79	NS	0.14
Mg	2800	56	3200	76	**	0.71	1800		20	1800	23	NS	0.21
Mn	150	4.4	200	5.8	***	0.55	5.5		0.12	6.1	0.14	**	0.39
Mo	0.4	0.009	0.36	0.007	*	0.31	0.072		0.007	0.052	0.009	NS	0.03
Na	10	0.48	8.2	0.39	NS	0.1	0.45		0.01	0.49	0.012	NS	0
Ni	0.037	0.0008	0.033	0.0007	NS	0.17	0.026		0.001	0.03	0.001	NS	0.46
P	2500	28	2300	40	**	0.63	2700		33	2800	43	NS	0.14
Rb	7.1	0.15	6.7	0.15	NS	0.4	1.7		0.05	1.6	0.055	NS	0
S	3800	32	3700	39	NS	0.51	2000		28	2000	25	NS	0.23
Se	0.018	0.0007	0.016	0.0006	NS	0	0.009		0.0003	0.008	0.0004	**	0
Sr	27	1	30	0.99	NS	0.66	0.43		0.018	0.52	0.023	NS	0.32
Zn	41	1	19	1.1	***	0.44	20		0.37	13	0.43	***	0.25

Means and standard error (S.E.) were calculated from 75 and 66 families for AMF-C and AMF-I, respectively. The significance of mean differences between two groups were tested using the Wilcoxon test with *p* values adjusted (Bonferroni adjustment) based on the number of elements

Broad sense heritability (H^2^) of the whole mapping population was estimated using the *bwardr::Cullis_H2* function for R

*NS* not significant

*p < 0.05; **p < 0.01; ***p < 0.001

AM symbiosis not only affected individual elements but also affected patterns of covariance among elements, with more than half of the significant correlations changing depending on AM status (Supplemental Dataset 1). There was a significant difference between the leaf element pairwise correlation matrices for AMF-C and AMF-I subpopulations (*p* < 0.001). For AMF-C and AMF-I leaves (Fig. 2C), 11 of the significant correlations were independent of AM status. In contrast, the sign of three correlations changed with AM status, indicating a strong effect of AMF on covariance (e.g. the positive correlation between Zn and the three elements Sr, Ca and Mn in AMF-C became negative in AMF-I). Ten significant correlations were specific to the AMF-C subpopulation and ten to the AMF-I subpopulation. The grain element correlation matrices, unlike those of leaves, were not significantly different between AMF-C and AMF-I subpopulations (*p* = 0.060), although only five pairwise correlations were shared across AM status (Fig. 2C).

### The genetic architecture of element concentration is modulated by AM incompatibility

**Fig. 3:**
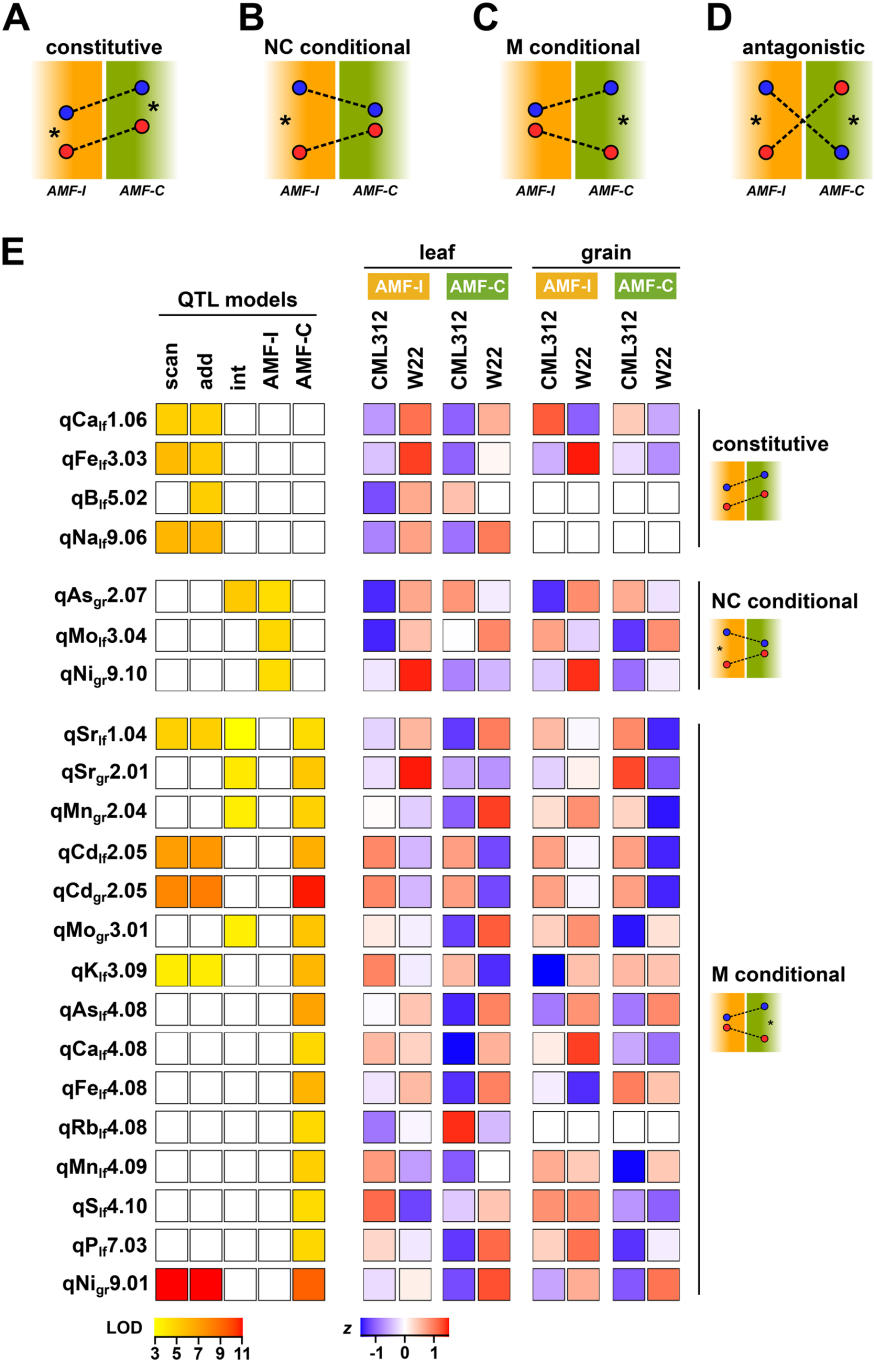
The behaviour of element QTLs is contingent on AM status. **A–D** Schematic plots of allele effects under different genetic scenarios as described in the ‘Introduction’ section. Vertical position of the points represents the relative trait value associated with two alleles at a given QTL in either AMF-I or AMF-C subpopulations. Dashed lines link the same allele across the two subpopulations. Asterisks indicate a significant allele effect for a given subpopulation. **E** QTL support (LOD) and allele effects (standardized *z* score for W22 or CML312 allele) for element QTLs identified by genetic mapping. QTLs are named for the associated element, the tissue type (lf for leaf or gr for grain) and the genetic position (chromosome and bin). Models labelled as *scan* for all families in a single QTL scan; *add* for AMF as an additive covariate; *int* for AMF as an interactive covariate; *AMF-I* for a separate analysis of the AMF-I subpopulation; *AMF-C* for a separate analysis of the AMF-C subpopulation. LOD support is shown only for models for which the QTL was significant. Effect estimates are shown for both leaf and grain and both AMF-I and AMF-C subpopulations irrespective of whether the QTL was significant in any given tissue/AMF combination. Effects were standardized separately for leaf and grain

Having quantified the main effect of AMF on the plant ionome, we proceeded to use Quantitative Trait Locus (QTL) mapping to identify genetic differences among families and to estimate GxAMF effects. By analogy to behaviour at the variety level, individual QTL may have the same effect in mycorrhizal or non-mycorrhizal plants (Fig. 3A), or the effect may be modified by AM symbiosis (i.e. a QTLxAMF effect; Fig. 3B, C). There was the additional possibility of a QTL being detected in both AMF-I and AMF-C subpopulations but with a change in the sign of the effect—a scenario known as *antagonistic pleiotropy* that, in this context, indicates a potential genetic trade-off between AMF and direct nutrient uptake (Fig. 3D; Des Marais and Juenger 2010; Perez-Limón et al. 2022; Ramírez-Flores et al. 2020).

To identify QTLs and assess QTLxAMF effects, we ran a series of QTL models: (1) both subpopulations together without taking AM status into account, (2) both subpopulations with AM status as an additive covariate, (3) both subpopulations with AM status as an interactive covariate, (4) AMF-I subpopulation alone and (5) AMF-C subpopulation alone. Model 2 eliminated the AMF main effect but did not allow for the QTL effect to differ with AM status (i.e. QTLxAMF effects are not modelled; the model describes well the scenario shown in Fig. 3A). QTLxAMF effects were modelled explicitly by model 3 and indirectly by comparison of the results of models 4 and 5. We considered leaf and grain element concentrations as separate traits in our analysis. Across all models, we detected a total of 22 element QTLs, considering QTLs for the same element detected in the same genetic bin in different models to be the same. When QTLs for different elements colocalized to the same bin, we report them as distinct loci, although they may represent a single pleiotropic genetic variant. QTLs were annotated by element, chromosome, genetic bin and tissue. Seven QTLs were detected for grain and 15 for leaves (Fig. 3E; Table 2; Supplemental Dataset 2). In the case of qCd_lf_2.05 and qCd_gr_2.05, QTLs for the same element co-localized in leaf and grain analyses likely representing a single causal variant, although we report them here as distinct. Across tissues, 18 QTLs showed evidence of QTLxAMF effects: fifteen QTLs detected specifically in the AMF-C subpopulation; three QTLs detected specifically in the AMF-I subpopulation. Five of the subpopulation-specific QTLs were further supported by Model 3. The QTLxAMF effects detected were all *conditional* (the scenarios shown in Fig. 3B, C), and we found no evidence of *antagonistic pleiotropy* (Fig. 3D). Globally, the sign of the allelic effect with respect to the two parents differed depending on the element (Fig. 3E). For example, for leaf QTLs detected in bin 4.08 in the AMF-C subpopulation, the CML312 allele was associated with a diminished concentration of Ca, Fe and As, but an elevated concentration of Rb. For qCd_lf/gr_2.05, the allelic effect was conserved across grain and leaves in the AMF-C subpopulation, the W22 allele being linked to a reduction in Cd concentration in both tissues (Figs. 3E and S2).

**Table 2:** Element QTLs detected in the analysis (α = 0.01)

**QTL name**	**Chr**	**Position (Mb)**	**Interval (Mb)**	**Model**	**QTL type**
qSr_Lf_1.04	1	53.68	27.8–201.66	scan, add, int, AMF-C, AMF-C	M
qCa_Lf_1.06	1	201.91	40.36–252.86	scan, add	C
qSr_Gr_2.01	2	5.43	3.12–5.43	int, AMF-C	M
qMn_Gr_2.04	2	45.83	17.55–50.58	int, AMF-C	M
qCd_Lf_2.05	2	64.91	28.62–155.24	scan, add, AMF-C	M
qCd_Gr_2.05	2	73.59	60.91–73.59	scan, add, AMF-C	M
qAs_Gr_2.07	2	190.28	187.09–192.03	int, AMF-I	NC
qMo_Gr_3.01	3	0.99	0.99–1.97	int, AMF-C	M
qFe_Lf_3.03	3	14.36	11.24–18.83	scan, add	C
qMo_Lf_3.04	3	148.01	138.81–158.93	AMF-I	NC
qK_Lf_3.09	3	221.66	216.61–223.84	scan, add, AMF-C	M
qAs_Lf_4.08	4	192.64	186.04–234.3	AMF-C	M
qCa_Lf_4.08	4	230.89	182.47–234.3	AMF-C	M
qFe_Lf_4.08	4	207.33	182.47–234.94	AMF-C	M
qRb_Lf_4.08	4	188.18	176.92–197.16	AMF-C	M
qMn_Lf_4.09	4	235.93	164.28–238.06	AMF-C	M
qS_Lf_4.10	4	237.69	237.69–239.75	AMF-C	M
qB_Lf_5.02	5	5.21	4.27–6.38	add	C
qP_Lf_7.03	7	10.17	3–165.63	AMF-C	M
qNi_Gr_9.01	9	2.49	2.49–4.29	scan, add, AMF-C	M
qNa_Lf_9.06	9	134.18	120.48–136.65	scan, add	C
qNi_Gr_9.10	9	150.52	2.49–153.19	AMF-I	NC

QTL name refers to the element, tissue, and genetic bin where the QTL was detected. Chr is the chromosome where the QTL was detected. Position and Interval report the physical coordinates (B73v3 genome) of the QTL peak and the 1.5 LOD-drop confidence interval, respectively. Model lists the QTL model/s in which the QTL was significant as *scan* for all families in a single QTL scan, *add* for AMF as an additive covariate, *int* for AMF as an interactive covariate, *AMF-I* for separate analysis of the AMF-I subpopulation and *AMF-C* for separate analysis of the AMF-C subpopulation. QTL type reports QTL as constitutive (C), mycorrhizal (M) conditional or non-colonized (NC) conditional

### QTL × AMF effects co-localized with QTL × E effects in a previous multisite experiment

**Fig. 4:**
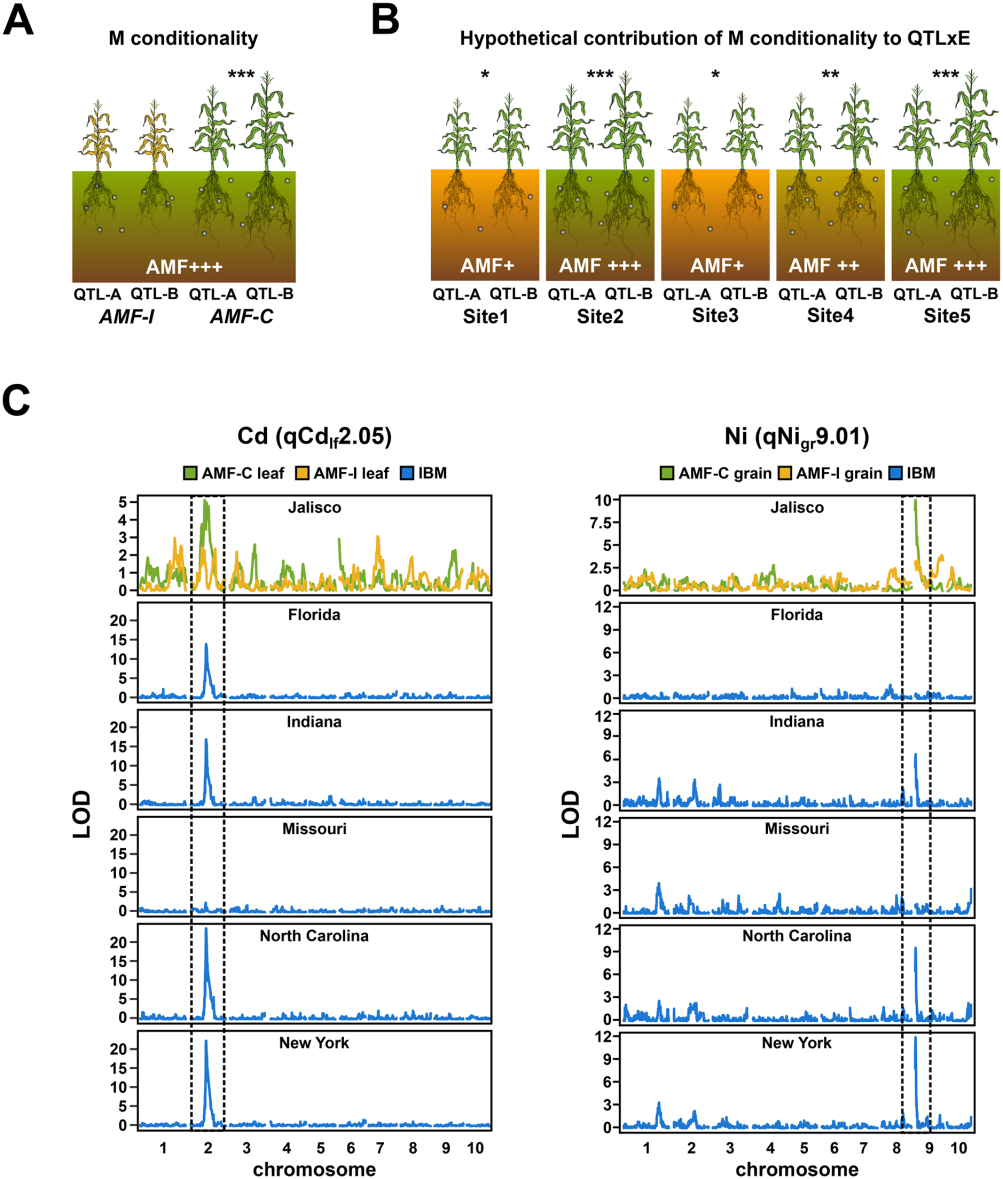
Mycorrhiza conditional Cd and Ni QTLs co-localize with corresponding QTLs showing environmental specificity in a previous multisite evaluation. **A** Behaviour of a hypothetical mycorrhizal (M) conditional QTL. In a field containing AMF, plants carrying allele A at a given QTL (QTL-A) differ phenotypically from those carrying allele B (QTL-B) when AMF-compatible (AMF-C) but not when AMF-incompatible (AMF-I). **B** Hypothetical contribution of M conditionality to QTL x environment interaction (QTLxE). In wild-type (AMF-C) plants grown across locations (Sites 1–5), the magnitude of the phenotypic difference between plants carrying QTL-A or QTL-B is contingent on the strength of the local AMF community. The number of asterisks indicates the magnitude of the QTL effect. The strength of the AMF community is shown from AMF+ to AMF+++. C) Overlap between M conditional QTL for Cd and Ni identified in the AMF-C/AMF-I experiment (Jalisco) and evaluation of the intermated B73 x Mo17 (IBM) mapping population across multiple sites (locations given by US state. Asaro et al. 2016). Plots show genome wide QTL support (LOD) associated with concentrations of the named elements in AMF and IBM experimental field data. IBM plots were averaged over years when multiple data were available. Dashed vertical lines highlight regions containing the named QTL identified in the AMF-C population

To obtain additional support for our QTLs, we compared our findings to a published maize multisite ionome dataset (Asaro et al. 2016). This previous work presented grain ionome data for a biparental maize mapping population (intermated B73 x Mo17, hereafter IBM), evaluated from 2005 to 2012, in five different US states—although without specific characterization of the soil microbial community. We re-analysed these data to facilitate comparison with our results, considering each location separately. We found clear overlap between our QTLs qCd_lf/gr_2.05 and qNi_gr_9.01 and corresponding signals from the IBM dataset (Table S1; Fig. 4). Interestingly, both qCd_lf/gr_2.05 and qNi_gr_9.01 M were conditional (Fig. 4A) in our experiments while showing evidence of QTL × E in the IBM study (Fig. 4C). Although difficult to substantiate without further data, we speculate that variation in the AMF community may have contributed to grain ionome differences between the IBM locations (Fig. 4B). For example, QTLs corresponding to qCd_lf/gr_2.05 and qNi_gr_9.01 were recovered in the IBM in North Carolina and New York but not in Missouri (Fig. 4C). Absolute element concentrations indicate that these elements were present in the Missouri environment. Given these QTL were M conditional in our study, we tentatively suggest that a weak AMF community in the Missouri site might have reduced their expression.

## Discussion

We have characterized the impact of AMF on mineral nutrition in field grown maize in a subtropical field site. Using a custom mapping resource comprising AMF-C and AMF-I maize subpopulations (for purposes of this discussion, we will generalize these as M and NC, respectively), we estimated the effect of AMF on the concentrations of 20 elements in the leaves and grain, evaluated the impact of AMF on covariance among elements and characterized the host genetic architecture of element concentrations with respect to host AM status.

In our trial, the concentrations of the essential nutrients Zn and Cu was greater in the grain of mycorrhizal plants, consistent with previous reported greenhouse studies (Lehmann and Rillig 2015; Lehmann et al. 2014). Treatments promoting increased grain yields in maize have been associated with a reduction in Zn concentration through a dilution effect (Zhang et al. 2021). In our experiment, however, M plants maintained a greater Zn concentration than NC plants even though these plants were previously reported to have a greater total grain weight than that of NC plants (Ramírez-Flores et al. 2020). P concentration was significantly elevated in the leaves, but not the grain, of M plants. We note, however, that both AMF-C and AMF-I subpopulations maintained an average P shoot concentration above a typical critical concentration (~ 2200 mg/kg; Gagnon et al. 2020), indicating that P was not limiting in this experiment. M plants showed a significant reduction in the concentration of the potentially toxic element As in both leaves and grain, again consistent with greenhouse observations (Göhre and Paszkowski 2006; Lehmann and Rillig 2015; Neidhardt 2021). The element As in the pentavalent form of arsenate (AsO_4_^3−^) is chemically similar to inorganic phosphate (PO_4_^3−^), and the two can compete for transport through the P uptake system (Meharg and Hartley-Whitaker 2002). AMF have been reported to reduce As uptake by downregulating transporters involved in the direct P uptake pathway (Christophersen et al. 2009; Li et al. 2018). We found that a significant negative correlation between P and As in the leaves of nonmycorrhizal plants was no longer significant in mycorrhizal plants, supporting an effect of AMF in changing the As-P relationship.

Alongside the largely desirable effects described above, we observed a potentially deleterious reduction in the concentration of the essential micronutrient Fe in both the leaves and grain of mycorrhizal plants. Fe reduction in the shoots of different greenhouse grown mycorrhizal plant species has been reported previously (Tran et al. 2019), although AMF also have been reported to specifically increase Fe concentration in roots (Watts-Williams and Cavagnaro 2014), suggesting a modulation of root-shoot translocation (Ibiang et al. 2017; Xie et al. 2019). The effect of AMF on Fe is further complicated by interactions with Zn. We observed a negative correlation between grain Zn and Fe concentrations in AMF-I families, in line with previously reported Zn-Fe antagonism in soybean shoots (Ibiang et al. 2017). In AMF-C families, however, the correlation between grain Zn and Fe concentration was positive. In addition to being essential for plants, Zn and Fe are the two elements most commonly lacking in human diets and major targets of biofortification breeding programs (Maqbool and Beshir 2019; White and Broadley 2009). In our population, the positive correlation between Zn and Fe concentration in mycorrhizal plants favours selection for a coordinate increase in the two elements, whereas the negative correlation in non-mycorrhizal plants would constrain selection along this trajectory.

We identified 18 element QTLs that showed evidence of interaction with AM status. All QTLxAMF effects were conditional, i.e. QTLs were specific to either M or NC plants, although limited statistical power may have prevented identification of more complex genetic architectures. Plant roots take up nutrients directly from the soil (the *direct* pathway) or acquire them via symbiosis with AMF (the *AMF* pathway; Smith et al. 2003). The contributions of direct and mycorrhizal uptake pathways are not simply additive but can act as alternative strategies, one or the other dominating under given conditions (Smith et al. 2003). Molecular analyses have supported this view through the identification of diversified families of plant nutrient transporters containing members that play specific roles in either the direct or the mycorrhizal uptake pathway and are regulated accordingly (Casieri et al. 2012; Hui et al. 2022; Koegel et al. 2013; Li et al. 2018; Yang et al. 2012). Our identification of conditional QTLs is consistent with variation in such pathway-specific genetic components, their effects being expressed preferentially in M or NC plants.

In addition to delivery of nutrients to the arbuscule, AMF secondarily influence nutrient uptake through modulation of root growth and development (Ramírez-Flores et al. 2019). As such, AM symbiosis has the capacity to either mask or to exaggerate variation in direct nutrient uptake among host genotypes, a further potential contribution to QTLxAMF effects. For example, we identified an M specific QTL linked to Cd concentration in leaf and grain (qCd_lf/gr_2.05). This QTL co-localized with a major locus for maize grain Cd concentration in a previous study that has been associated with the heavy metal transporter ZmHMA3a (Chen et al. 2022; Tang et al. 2021). AMF previously have been reported to upregulate the expression of the orthologous transporter gene in rice and thus to promote sequestration of Cd in vacuoles of root cells and to reduce the translocation of Cd from roots to shoots (Chen et al. 2019; Zhu et al. 2022). Our results suggest that the efficacy of this protection is itself contingent on the host genetic background.

The co-localization of AMF-conditional QTL and those identified in a grain ionome study carried out across multiple years and locations (Asaro et al. 2016) supports the authenticity of these signals. Furthermore, the association of M conditionality and QTL × E in the previous work is consistent with the hypothesis that variation in AMF communities is contributing to G × E effects. Broadly considered, the contribution of GxAMF is relevant to any proposition to best align agricultural systems to benefit from AMF. In the absence of GxAMF, an improved AMF community (however promoted, e.g. by reduced synthetic inputs, reduced tillage, application of biofertilizers) will benefit all varieties equally with no need to consider the plants themselves. However, if, as our data suggest, the impact of AMF is conditional on the host genotype, we cannot suppose that crop varieties optimized, for example, in an AMF repressive environment will necessarily obtain the greatest benefit when a stronger AMF community is present. The size of the AMF effect and the relative importance of GxAMF interactions will change with environment (including agricultural management) and the plant material under consideration. In this study, we have illustrated an approach that has the potential to improve our estimation of this key aspect of the role of AMF in cultivated fields.

### Supplementary Information

Below is the link to the electronic supplementary material.

Supplementary file1 (XLSX 32 KB)

Supplementary file2 (CSV 4 KB)

Supplementary file3 (PDF 255 KB)

## Data Availability

Genotype and phenotype data and supplementary information are provided on Figshare under the doi’s: https://doi.org/10.6084/m9.figshare.21684947.

## Declarations

### Completing interests

The authors declare no completing interests.
