# Supplementary material for: Mycorrhizal status and host genotype interact to shape plant nutrition in field grown maize (Zea mays ssp. mays)
Source: Mycorrhiza. 2023 Oct 18;33(5-6):345–58. doi: 10.1007/s00572-023-01127-3 (PMC10752836; doi:10.1007/s00572-023-01127-3)
Supplement: Supplementary file 3 — Supplementary file3 (PDF 255 KB) [file 572_2023_1127_MOESM3_ESM.pdf]

Supplementary Table S1. QTLs detected in common with a published multisite ionome analysis (Asaro *et al.*, 2016). Significance threshold is alpha = 0.1. QTLs are named by trait, tissue and genomic bin, and Chr is the chromosome number. Asaro Trait represents the phenotype in the published multisite ionome analysis (Asaro *et al.*, 2016). AMF_pos is the position of the AMF QTL in the CML312xW22 genetic map (cM). Est_AMF_pos is the estimated position of the QTL detected in Asaro's work in CML312xW22 genetic coordinates (cM). AMF physical interval is the 1.5 LOD support QTL interval detected in this study in the B73 v3 physical position (MB). Asaro physical interval is the 1.5 LOD support QTL interval detected in Asaro's work in the B73 v3 physical position (MB).

QTL	Asaro Trait	Chr	AMF_pos	Est_AMF_pos	AMF physical interval	Asaro physical interval
qCd_Lf_2.05	Cd	2	58.5	64	28.5 - 155	151.43 - 165.89
qNi_Gr_9.01	Ni	9	1	2.05	2.49 - 4.29	2.49 – 4.29

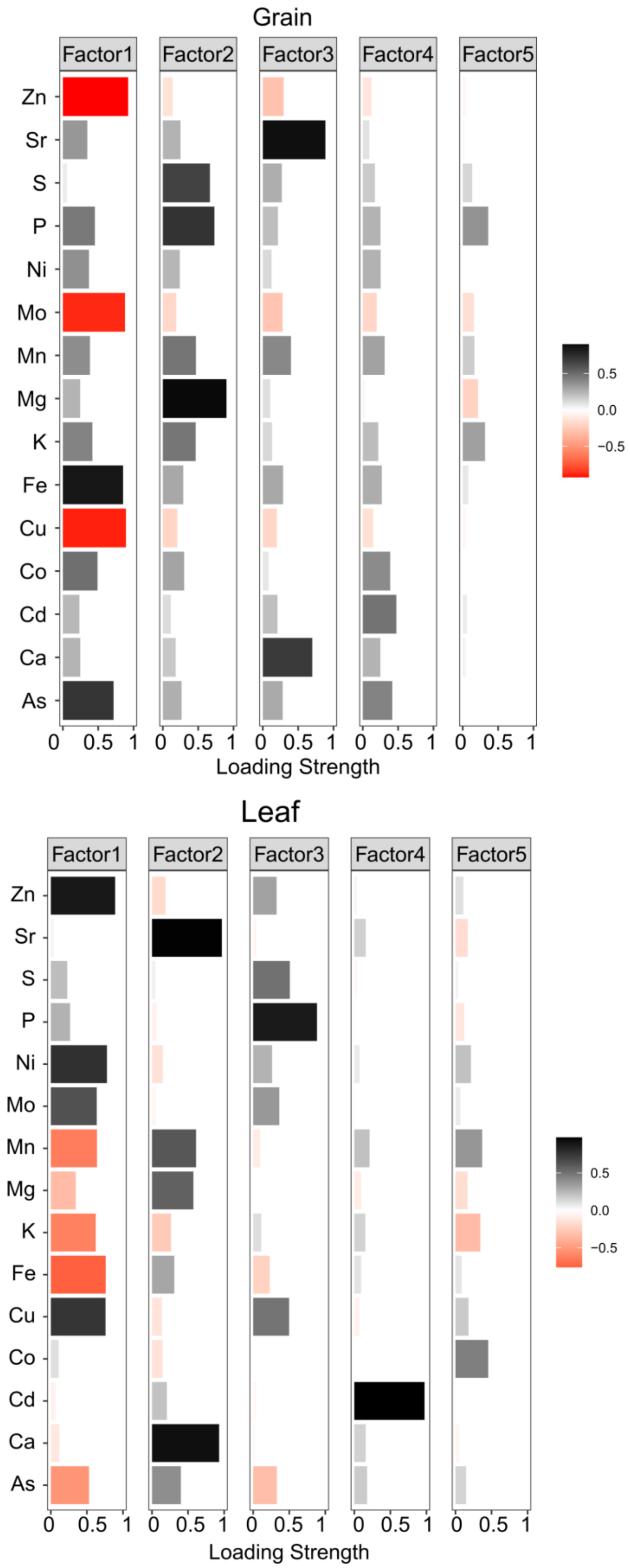

Fig. S1. Loadings showing the contributions of elements to the first five factors of the Factor Analysis of element contractions in leaf and grain.

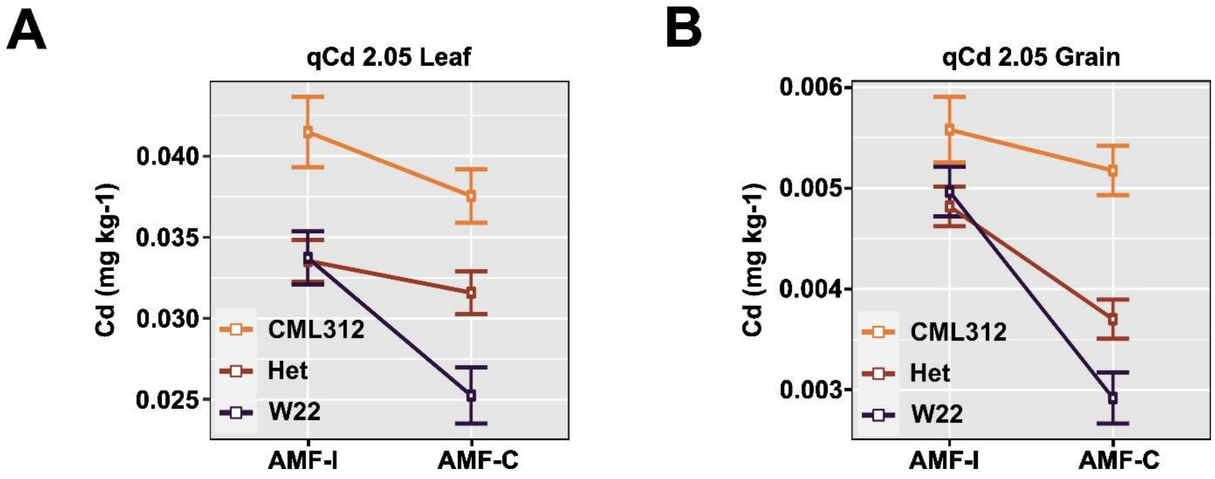

Fig. S2. **Effect of QTL linked to cadmium concentration.** A, B) Effect of $qCd_{lf/gr}$ 2.05 in leaves and grain, respectively. Plots show the fitted estimated Cd concentration (mg kg^{-1} +/- 1SE) for the three genotype classes at the QTL (homozygous CML312; heterozygous CML312/W22; homozygous W22) in AMF incompatible (AMF-I) an AMF-compatible (AMF-C) subpopulations.
